# 3-Amino-1-methyl­pyrazin-1-ium chloride

**DOI:** 10.1107/S1600536809051265

**Published:** 2009-12-04

**Authors:** Daniel Foucher, Stephen Wylie, Joshua Acosta, Alan J. Lough

**Affiliations:** a Department of Chemistry and Biology, Ryerson University, Toronto, Ontario, Canada, M5B 2K3; b Department of Chemistry, University of Toronto, Toronto, Ontario, Canada, M5S 3H6

## Abstract

In the cation of the title compound, C_5_H_8_N_3_^+^·Cl^−^, the C—N(H_2_) bond distance [1.348 (3) Å] is at the lower end of the range for aryl amines. In the crystal structure, cations and anions are linked *via* N—H⋯Cl hydrogen bonds, forming one-dimensional chains along [100].

## Related literature

For the synthesis and characterization of the title compound, see: Foucher *et al.* (1993 ▶). Additional preparative details of similar compounds are given by Goto *et al.* (1968 ▶). For related structures, see Chao *et al.* (1976 ▶); Kazheva *et al.* (2006 ▶); Foucher *et al.* (1989 ▶); Lu & Xi (2008 ▶). For the crystal structure of 3-amino-1- methylpyrazin-1-ium iodide, see: Foucher *et al.* (2009 ▶). For comparative bond-distance data, see: Allen *et al.* (1987 ▶).
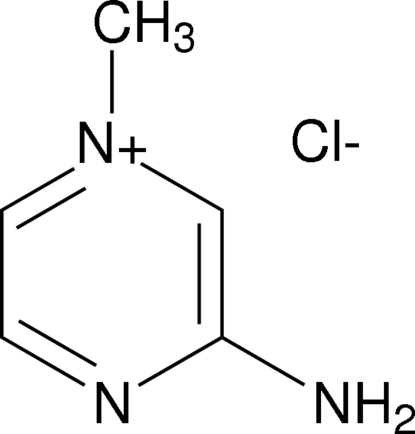


## Experimental

### 

#### Crystal data

C_5_H_8_N_3_^+^·Cl^−^
*M* *_r_* = 145.59
Orthorhombic, 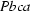

*a* = 11.3164 (3) Å
*b* = 9.5029 (5) Å
*c* = 12.3877 (5) Å
*V* = 1332.16 (10) Å^3^
*Z* = 8
Mo *K*α radiation
μ = 0.48 mm^−1^
*T* = 150 K
0.24 × 0.16 × 0.12 mm

#### Data collection

Nonius KappaCCD diffractometer
Absorption correction: multi-scan (*SORTAV*; Blessing 1995 ▶) *T*_min_ = 0.819, *T*_max_ = 0.946
9107 measured reflections
1526 independent reflections
1144 reflections with *I* > 2σ(*I*)
*R*_int_ = 0.047

#### Refinement

*R*[*F* ^2^ > 2σ(*F* ^2^)] = 0.040
*wR*(*F* ^2^) = 0.111
*S* = 1.10
1526 reflections
91 parameters
H atoms treated by a mixture of independent and constrained refinement
Δρ_max_ = 0.46 e Å^−3^
Δρ_min_ = −0.28 e Å^−3^

### 

Data collection: *COLLECT* (Nonius BV, 2002 ▶); cell refinement: *DENZO-SMN* (Otwinowski & Minor, 1997 ▶); data reduction: *DENZO-SMN*; program(s) used to solve structure: *SIR92* (Altomare *et al.*, 1994 ▶); program(s) used to refine structure: *SHELXTL* (Sheldrick, 2008 ▶); molecular graphics: *PLATON* (Spek, 2009 ▶); software used to prepare material for publication: *SHELXTL*.

## Supplementary Material

Crystal structure: contains datablocks global, I. DOI: 10.1107/S1600536809051265/tk2586sup1.cif

Structure factors: contains datablocks I. DOI: 10.1107/S1600536809051265/tk2586Isup2.hkl

Additional supplementary materials:  crystallographic information; 3D view; checkCIF report

## Figures and Tables

**Table 1:** Hydrogen-bond geometry (Å, °)

*D*—H⋯*A*	*D*—H	H⋯*A*	*D*⋯*A*	*D*—H⋯*A*
N7—H1*N*⋯Cl1	0.91 (3)	2.40 (3)	3.297 (2)	168 (2)
N7—H2*N*⋯Cl1^i^	0.94 (3)	2.37 (3)	3.289 (2)	168 (3)

Symmetry code: (i) 
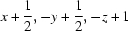
.

**Fig. 1.**
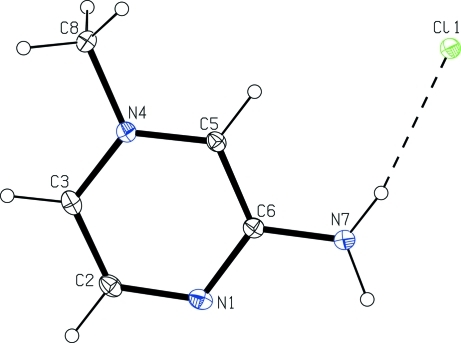
The asymmetric unit of (I) with displacement ellipsoids drawn at the 30% probability level. The dashed line indicates a hydrogen bond.

**Fig. 2.**
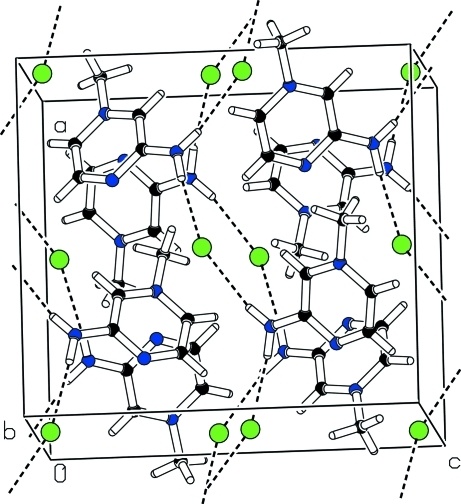
Part of the crystal structure of (I) with hydrogen bonds shown as dashed lines.

## supplementary crystallographic information

### Comment

The title chloride compound, (I), was recovered from the ion exchange (Dowex 1-X8 ion exchange resin saturated with Cl^-^ anions) of the iodide precursor of *N*-methyl-3-aminopyrazinium iodide (Foucher *et al.*, 1993). The proximity of the amine group to one of the diazine N atoms makes it an ideal chelating ligand to metals and geometrically suggests the possibility for amine-imine tautomerism. We have investigated the possibility that a smaller counter ion might induce a preference for the imine tautomer in these salts.

The molecular structure of (I) is shown in Fig. 1. The cation is the amine tautomer and resembles closely in terms of bond angles and bond lengths, other *N*-methylated amino pyrazinium salts (Kazheva *et al.*, 2006; Foucher *et al.*, 1989). The C5—N4—C3 bond angle in (I) [121.02 (18) °] is significantly wider than in 2-aminopyrazine [116.6 (1)°] (Chao *et al.*, 1976) but similar to the angle found in *N*-methyl-3-aminopyrazinium iodide (121.3 (5)°; Foucher *et al.*, 2010). 2-Aminopyrazine and both *N*-methyl-3-aminopyrazium salts are characterized by short amine-ring bond distances [N7—C6 in (I) = 1.348 (3) Å, 1.341 (1)Å (Chao *et al.*, 1976) and 1.338 (8)Å (Foucher *et al.*, 2009)] compared to typical values for C(*sp*^2^)-NH_2_ bond lengths, *i.e.* 1.36 Å (Allen *et al.*, 1987)] although these distances are significantly longer than the C=N(H) bond [1.285 (4) Å] in *N*-(4-imino-3,5-dimethylcyclohexa-2,5-dienylidene)-2,6-dimethylaniline (Lu & Xi, 2008). These short bond lengths are suggestive of a considerable degree of double bond character, where the lone pair of the amine participates in the resonance of the ring π system. In the crystal structure, cations and anions are linked *via* intermolecular N—H···Cl hydrogen bonds to form one-dimensional chains along [100], Table 1 and Fig. 2.

### Experimental

General procedures for the synthesis of this type of compound are given by Goto *et al.* (1968) and Kazheva *et al.* (2006). The title compound was recovered from the ion exchange (Dowex 1-X8 ion exchange resin saturated with Cl^-^ anion) of a concentrated aqueous solution containing 0.30 g (1.266 mmol) of *N*-methyl-3-pyrazinium iodide (Foucher *et al.*, 1993). The aqueous fractions containing the crude title compound were collected and brought to dryness. Crystals suitable for X-ray diffraction were isolated from the recrystallization of the crude product from boiling ethanol. Yield 0.11 g, 78%. Characterization by NMR agreed with previous literature (Foucher *et al.*, 1993).

### Refinement

H atoms bonded to C atoms were placed in calculated positions with C—H = 0.95 and 0.98 Å, and included in a riding-model approximation with *U*_iso_(H) = 1.2*U*_eq_(C) or 1.5*U*_eq_(C_methyl_). H atoms bonded to the amine group N atom were refined independently with isotropic displacement parameters.

### Crystal data

**Table:**

C_5_H_8_N_3_^+^·Cl^−^	*F*(000) = 608
*M**_r_* = 145.59	*D*_x_ = 1.452 Mg m^−^^3^
Orthorhombic, *P**b**c**a*	Mo *K*α radiation, λ = 0.71073 Å
Hall symbol: -P 2ac 2ab	Cell parameters from 9107 reflections
*a* = 11.3164 (3) Å	θ = 3.3–27.5°
*b* = 9.5029 (5) Å	µ = 0.48 mm^−^^1^
*c* = 12.3877 (5) Å	*T* = 150 K
*V* = 1332.16 (10) Å^3^	Needle, pale yellow
*Z* = 8	0.24 × 0.16 × 0.12 mm

### Data collection

**Table:**

Nonius KappaCCD diffractometer	1526 independent reflections
Radiation source: fine-focus sealed tube	1144 reflections with *I* > 2σ(*I*)
graphite	*R*_int_ = 0.047
Detector resolution: 9 pixels mm^-1^	θ_max_ = 27.5°, θ_min_ = 3.3°
φ scans and ω scans with κ offsets	*h* = −14→14
Absorption correction: multi-scan (*SORTAV*; Blessing 1995)	*k* = −11→12
*T*_min_ = 0.819, *T*_max_ = 0.946	*l* = −16→15
9107 measured reflections	

### Refinement

**Table:**

Refinement on *F*^2^	Primary atom site location: structure-invariant direct methods
Least-squares matrix: full	Secondary atom site location: difference Fourier map
*R*[*F*^2^ > 2σ(*F*^2^)] = 0.040	Hydrogen site location: inferred from neighbouring sites
*wR*(*F*^2^) = 0.111	H atoms treated by a mixture of independent and constrained refinement
*S* = 1.10	*w* = 1/[σ^2^(*F*_o_^2^) + (0.0514*P*)^2^ + 0.7939*P*] where *P* = (*F*_o_^2^ + 2*F*_c_^2^)/3
1526 reflections	(Δ/σ)_max_ < 0.001
91 parameters	Δρ_max_ = 0.46 e Å^−^^3^
0 restraints	Δρ_min_ = −0.28 e Å^−^^3^

### Special details

**Table:**

Geometry. All e.s.d.'s (except the e.s.d. in the dihedral angle between two l.s. planes) are estimated using the full covariance matrix. The cell e.s.d.'s are taken into account individually in the estimation of e.s.d.'s in distances, angles and torsion angles; correlations between e.s.d.'s in cell parameters are only used when they are defined by crystal symmetry. An approximate (isotropic) treatment of cell e.s.d.'s is used for estimating e.s.d.'s involving l.s. planes.
Refinement. Refinement of *F*^2^ against ALL reflections. The weighted *R*-factor *wR* and goodness of fit *S* are based on *F*^2^, conventional *R*-factors *R* are based on *F*, with *F* set to zero for negative *F*^2^. The threshold expression of *F*^2^ > σ(*F*^2^) is used only for calculating *R*-factors(gt) *etc*. and is not relevant to the choice of reflections for refinement. *R*-factors based on *F*^2^ are statistically about twice as large as those based on *F*, and *R*- factors based on ALL data will be even larger.

### Fractional atomic coordinates and isotropic or equivalent isotropic displacement parameters (Å^2^)

**Table:**

	*x*	*y*	*z*	*U*_iso_*/*U*_eq_	
Cl1	0.52068 (5)	0.20079 (5)	0.55295 (4)	0.02322 (19)	
N1	0.80709 (16)	0.15734 (19)	0.21473 (15)	0.0255 (4)	
C2	0.7861 (2)	0.0803 (2)	0.12664 (17)	0.0257 (5)	
H2A	0.8438	0.0795	0.0710	0.031*	
C3	0.68473 (19)	0.0018 (2)	0.11251 (17)	0.0256 (5)	
H3A	0.6730	−0.0519	0.0487	0.031*	
N4	0.60290 (15)	0.00317 (17)	0.19137 (13)	0.0202 (4)	
C5	0.61964 (18)	0.0768 (2)	0.28134 (16)	0.0207 (5)	
H5A	0.5618	0.0767	0.3369	0.025*	
C6	0.72478 (18)	0.1552 (2)	0.29291 (17)	0.0212 (5)	
N7	0.74454 (18)	0.2293 (2)	0.38395 (16)	0.0301 (5)	
C8	0.49086 (19)	−0.0728 (2)	0.17540 (19)	0.0256 (5)	
H8A	0.4646	−0.1126	0.2443	0.038*	
H8B	0.5026	−0.1488	0.1231	0.038*	
H8C	0.4308	−0.0075	0.1482	0.038*	
H1N	0.691 (2)	0.227 (3)	0.438 (2)	0.035 (7)*	
H2N	0.821 (3)	0.263 (3)	0.397 (2)	0.058 (9)*	

### Atomic displacement parameters (Å^2^)

**Table:**

	*U*^11^	*U*^22^	*U*^33^	*U*^12^	*U*^13^	*U*^23^
Cl1	0.0224 (3)	0.0264 (3)	0.0209 (3)	−0.0013 (2)	0.0005 (2)	−0.00014 (19)
N1	0.0213 (9)	0.0295 (9)	0.0257 (10)	0.0032 (8)	0.0023 (8)	0.0047 (8)
C2	0.0255 (11)	0.0306 (11)	0.0210 (11)	0.0062 (9)	0.0037 (9)	0.0048 (9)
C3	0.0299 (12)	0.0267 (11)	0.0202 (11)	0.0069 (9)	0.0027 (9)	0.0000 (9)
N4	0.0211 (9)	0.0195 (8)	0.0199 (9)	0.0032 (7)	−0.0003 (7)	0.0014 (7)
C5	0.0206 (10)	0.0225 (10)	0.0191 (11)	0.0037 (8)	0.0011 (8)	0.0008 (8)
C6	0.0206 (11)	0.0218 (10)	0.0212 (11)	0.0029 (8)	−0.0004 (8)	0.0025 (8)
N7	0.0209 (10)	0.0421 (12)	0.0272 (11)	−0.0047 (9)	0.0017 (9)	−0.0085 (9)
C8	0.0241 (11)	0.0273 (11)	0.0254 (12)	−0.0029 (9)	−0.0020 (9)	−0.0047 (9)

### Geometric parameters (Å, °)

**Table:**

N1—C2	1.335 (3)	C5—C6	1.411 (3)
N1—C6	1.344 (3)	C5—H5A	0.9500
C2—C3	1.380 (3)	C6—N7	1.348 (3)
C2—H2A	0.9500	N7—H1N	0.91 (3)
C3—N4	1.346 (3)	N7—H2N	0.94 (3)
C3—H3A	0.9500	C8—H8A	0.9800
N4—C5	1.330 (3)	C8—H8B	0.9800
N4—C8	1.472 (3)	C8—H8C	0.9800
			
C2—N1—C6	117.23 (18)	N1—C6—N7	118.70 (19)
N1—C2—C3	123.2 (2)	N1—C6—C5	121.26 (19)
N1—C2—H2A	118.4	N7—C6—C5	120.03 (19)
C3—C2—H2A	118.4	C6—N7—H1N	119.8 (16)
N4—C3—C2	118.34 (19)	C6—N7—H2N	118.6 (19)
N4—C3—H3A	120.8	H1N—N7—H2N	120 (2)
C2—C3—H3A	120.8	N4—C8—H8A	109.5
C5—N4—C3	121.02 (18)	N4—C8—H8B	109.5
C5—N4—C8	119.57 (17)	H8A—C8—H8B	109.5
C3—N4—C8	119.35 (18)	N4—C8—H8C	109.5
N4—C5—C6	118.90 (19)	H8A—C8—H8C	109.5
N4—C5—H5A	120.6	H8B—C8—H8C	109.5
C6—C5—H5A	120.6		

### Hydrogen-bond geometry (Å, °)

**Table:**

*D*—H···*A*	*D*—H	H···*A*	*D*···*A*	*D*—H···*A*
N7—H1N···Cl1	0.91 (3)	2.40 (3)	3.297 (2)	168 (2)
N7—H2N···Cl1^i^	0.94 (3)	2.37 (3)	3.289 (2)	168 (3)

Symmetry codes: (i) *x*+1/2, −*y*+1/2, −*z*+1.
